# Association between e-health literacy and perceived importance of future pandemic preparedness in sub-saharan Africa

**DOI:** 10.1038/s41598-024-80121-x

**Published:** 2024-12-28

**Authors:** Emery Manirambona, Naimah Ebrahim Khan, Oluwabunmi Ogungbe, Sarah Irakoze, Jiaying Li, Emmanuel Uwiringiyimana, Israel Opeyemi Fawole, Cyriaque Habarugira, Oluwadamilare Akingbade, Aimable Nzabonimana, Oluwadamilola Agnes Fadodun, Madeleine Mukeshimana, Daniel YT Fong, Samuel Byiringiro

**Affiliations:** 1 College of Medicine and Health Sciences, University of Rwanda, Kigali, Rwanda; 2 Department of Biological and Medical Sciences, Faculty of Health and Life Sciences, Oxford Brookes University, Oxford, UK; 3 Department of Optometry, University of KwaZulu-Natal, Durban, South Africa; 4 School of Nursing, Johns Hopkins University, Baltimore, MD USA; 5 Department of Reanimation, Kamenge University Military Hospital, Bujumbura, Burundi; 6 School of Nursing, Li Ka Shing Faculty of Medicine, University of Hong Kong, Pok Fu Lam, Hong Kong SAR; 7 Research and Statistics, Institute of Nursing Research, Osogbo, Nigeria; 8 Hakipa Men’s Clinic, Bujumbura, Burundi; 9 Faculty of Nursing, University of Alberta, Edmonton, Canada; 10 Center for Language Enhancement, College of Arts and Social Sciences, University of Rwanda, Kigali, Rwanda; 11 Alberta Health Services, Elk Point, Canada; 12 School of Nursing and Midwifery, College of Medicine and Health Sciences, University of Rwanda, Kigali, Rwanda; 13 School of Nursing, The University of Hong Kong, Hong Kong, China

**Keywords:** Communicable diseases, Infection prevention & control, Disease outbreak response, Pandemics preparedness, E-Health literacy, Cross-sectional studies, Telemedicine, Community interventions, Sub-saharan Africa, Health care, Medical research

## Abstract

**Introduction:**

Emerging and re-emerging infectious diseases continue to pose a severe threat to public health in Sub-Saharan Africa (SSA) and globally. Community-related interventions, such as community e-Health literacy, can contribute to the preparedness to respond effectively to emerging and re-emerging infectious diseases. This study investigated the relationship between e-Health literacy and SSA countries’ perceptions of the importance of readiness for potential pandemics.

**Method:**

This cross-sectional study was conducted in sub-Saharan African countries (Nigeria, Rwanda, Burundi, and South Africa) among adults aged 18 years and above between July 2020 and August 2021, respondents were recruited through a non-probability sampling technique. Participants were asked to self-report the perceived importance of 13 items on future pandemic preparedness scored on a 5 Likert-point scale. The four key dimensions of pandemic preparedness were online medical consultation, online courses, messaging for healthcare, and shopping. E-Health literacy was the key exposure. The questionnaire was adapted from a previously validated e-Health literacy scale. Data was collected through a self-administered questionnaire online. Data analysis was done using Stata and descriptive statistics including frequency, proportions, means, and standard deviation were used to summarize variables. Inferential statistics including chi-square and logistic regressions were used to test the significance of association between e-health literacy and pandemic preparedness setting the level of significance at 5%.

**Results:**

A total of 1295 people participated in this study. Roughly half of all participants, 685 (52.90%), were aged between 18 and 29 and 685 (52.90%) were females. The standardised average (SE) e-Health literacy score was 29.55 (0.19). Shopping was perceived as the most important dimension of pandemic preparedness across participating countries (mean (SE) of 3.32 (0.06) and above across all countries for online shopping), while online medical consultation was the least perceived as important (mean (SE) of 2.88 (0.08) or less in two countries for instant health advice from chatbot). In the fully adjusted model, e-Health literacy was associated with 8 out of 13 items of the perceived importance of the pandemic preparedness questionnaire. Those include online consultation with doctors (OR = 1.11, 95% CI 1.02–1.21), telephone health advice (OR = 1.07, 95%CI 1.00–1.15), medicine delivery (OR = 1.04, 95% CI 1.03–1.06), getting medicine prescribed in a hospital visit/follow-up in a community pharmacy (OR = 1.07, 95% CI 1.05–1.10), receiving health information via email (OR = 1.08, 95% CI 1.01–1.17) and via social media (OR = 1.08, 95% CI 1.03–1.14), online shopping (OR = 1.07, 95% CI 1.03–1.11) and instant streaming courses (OR = 1.09, 95% CI 1.02–1.16).

**Conclusions:**

The higher e-Health literacy scores were associated with a higher perception of most elements as important in future pandemic readiness. Strengthening e-Health literacy can be a key element of the preparation for pandemics in SSA countries.

**Supplementary Information:**

The online version contains supplementary material available at 10.1038/s41598-024-80121-x.

## Introduction

Emerging infectious disease (EID) outbreaks with epidemic potential are a global health threat in the 21st century^1^. For instance, according to the World Health Organization data, over 776,007,137 people caught COVID-19, and close to 7,059,612 lives were lost to the COVID-19 pandemic as of 18 August 2024 ^2^. In Africa, the threats from emerging and re-emerging infectious diseases are not new. Research on Egyptian human remains has shown that Tuberculosis and Schistosomiasis killed people for at least three millennia^3,4^. Other additional emerging and re-emerging infectious diseases were documented in Sub-Saharan Africa (SSA) in the last century and they include Rift Valley Fever, first documented in Kenya in 1931, Zika in Uganda in 1947, Chikungunya in Tanzania in 1952, Lassa Fever in Nigeria in 1969, and Monkeypox (Mpox) and Ebola virus disease in the Democratic Republic of the Congo in 1970 and 1976, respectively^5^.

The spread of emerging and re-emerging infectious diseases has evolved, and multiple factors are responsible for that change. For instance, certain zoonotic infections originate from wildlife, while others come from non-wildlife sources^6^. Additionally, certain categories of pathogens were brought on by vector-borne or drug-resistant pathogens^6^. Viral factors facilitate the quick evolution of the viruses and the viral adaptation to the environment, allowing their Ribonucleic Acid (RNA) to reach the host quickly while maintaining balance^7^. In addition, ecological factors such as fluctuations in rainfall patterns, frequent droughts, floods, and other extreme climatic events partially resulting from the increase in global warming can contribute to the spread of the emerging and re-emerging infectious diseases^7^. Moreover, environmental disasters can destroy essential infrastructure, thus leading to poor sanitation and precarious hygienic conditions, further facilitating the spread of emerging and re-emerging infectious diseases^8^.

Human factors are another major driver of the increased spread of EIDs^7^. The rise in global and in-country movement of people is associated with population growth and technology, which increases the transmission of emerging and re-emerging infectious diseases^7^.The connection to this is urbanisation that fosters overcrowded conditions, leading to poor water, sanitation, and hygiene (WASH) and non-adherence to preventive practices such as social distancing. Of note, human social behaviour and activities such as unprotected sexual intercourse further increase the spread of some EIDs, and the injudicious use of antiviral drugs, antibiotics misuse, and deforestation may contribute to the evolution of the pathogens or resistance of strains or break zoonotic transmission cycle, thus increasing the disease transmission and pathogenicity^1,6^. The Mpox outbreak and COVID-19 are recent examples of emerging and re-emerging infectious diseases whose spread was exacerbated by human factors. This should stress the need to be prepared to handle pandemics effectively.

The readiness to efficiently respond to epidemics and pandemics is associated with community-related interventions that include but are not limited to community health literacy^9^. Health literacy, defined by the World Health Organisation (WHO) as the ability to gain access to information and understand its use for health promotion, is essential to sustaining good health outcomes^10^. In today’s era when most information is stored and distributed over internet, electronic health (e-Health) literacy, which encompasses finding appropriate health information from electronic sources, critically appraising it, and applying it to take health actions, is utterly critical^11^. This means that e-Health literacy can play an important role in sharing health information and transferring health-related knowledge necessary to devise health policies for population health promotion^11,12^.

Furthermore, e-Health literacy can play a significant role in emerging and re-emerging infectious diseases management^13^. The ubiquity of internet use does facilitate the sharing of health information but also leads to a quicker spread of misinformation, which can occasionally be extremely harmful. A higher level of e-Health literacy would help individuals sieve through website information and identify credible from misleading information sources, leading to positive health behaviour changes^11^. E-Health literacy has shown to be essential in providing necessary knowledge thus enhancing compliance with the devised COVID-19 preventive behaviours, including vaccination^11,13^. Further, e-Health literacy can make it easy to engage in activities such as telemedicine, online learning, and shopping for goods and medications, which often become essential to have in the instances of social contact restrictions in early and sometimes ongoing management of EID outbreaks^14^.

A prepared community is key to a timely and effective response to a pandemic, thus curbing the negative health and socio-economic outcomes often associated with emerging and re-emerging infectious diseases^15^. Therefore, it is critical to analyse and address the community’s perception regarding the future pandemic preparedness. However, the association between e-Health literacy and perception of future pandemic preparedness has not yet been explored. Accordingly, this research aimed to investigate the association between e-Health literacy and the perception of future pandemic preparedness in SSA.

## Method

### Study design and setting

We used a cross-sectional design to explore the association between e-Health literacy and perceived future pandemic preparedness in four SSA countries (Nigeria, Rwanda, Burundi, and South Africa). We followed the STROBE (Strengthening the Reporting of Observational Studies in Epidemiology) checklist to ensure the completeness and accuracy of this study^16^.

### Study population and sample

During the COVID-19 pandemic, we launched an international survey assessing the impact of COVID-19 on fear and health (CARE)^17^. We used a non-probability sampling technique as it was cost-effective and easily accessible in the context of the COVID-19 pandemic social distancing restrictions during the data collection timeframe. People aged 18 years and above, capable of providing consent, were eligible to participate in the study and participation was voluntary. In each country, a target of 500 respondents by country was sought, even though this goal was achieved in some but not all countries. A total of XXX individuals from XXX countries across XX continents participated in CARE. In Sub-Saharan Africa, Nigeria, Rwanda, Burundi, and South Africa participated in CARE. The current analysis focused on Sub-Saharan African countries because of their unique challenges related to health literacy and a disparately higher burden of infectious diseases. Detailed methods of the CARE project were published elsewhere^17^.

### Recruitment, data collection tool and data collection

#### Recruitment

The recruitment for the CARE project was essentially based on social media and other online platforms, including email and Short Messaging Systems (SMS). The social media platforms utilised in SSA for recruitment and distribution of the survey include WhatsApp, FaceBook, LinkedIn, Twitter, and Instagram. All participants were encouraged to share the online survey widely to their social networks. The recruitment and data collection for CARE in SSA occurred between July 2020 and August 2021.

#### Data collection tool

The questionnaire was divided into two main sections that include e-HEALS and perceived importance of pandemic preparedness.

The e-HEALS section was adapted or adopted from a previously validated e-health literacy scale^18^. The e-HEALS has 8 items: #1. I know how to find helpful health resources on the Internet, #2. I know how to use the Internet to answer my questions about health, #3. I know what health resources are available on the Internet, #4. I know where to find helpful health resources on the Internet, #5. I know how to use the health information I find on the Internet to help me, #6. I have the skills I need to evaluate the health resources I find on the Internet, #7. I can tell high-quality health resources from low-quality health resources on the Internet, and #8. I feel confident in using information from the Internet to make health decisions.

We developed 13 items to evaluate and assess perceived importance of pandemic preparedness as there were no validated questionnaires available. The 13 items, as described below, were discussed and agreed by collaborators from all participating countries aiming the comparison of participants’ preferences in the line of individual preparedness. These 13 items were about #1. Online consultation with doctors (e.g. Zoom, Skype), #2. Instant personalised health advice by online chatbot, #3. Telephone health advice, #4. Online courses, #5. Instant streaming courses (e.g. Zoom, Skype), #6. Receiving health information through email, #7: Receiving health information through text messaging (e.g. SMS, WhatsApp), #8: Receiving health information from social media (e.g. Facebook, Instagram, Twitter), #9. Receiving health information from a mobile app, #10: Get medicine prescribed in a hospital visit/follow-up in a community pharmacy, #11: Medicine delivery, #12. Online shopping, and #13. Food delivery.

#### Data collection method

The survey was self-administered online and consisted of a battery of questionnaires, which took approximately 30 min to complete. Where necessary, the survey was translated from English to the local language. In Sub-Saharan Africa, the survey was administered in English (Nigeria, South Africa, Burundi, and Rwanda) and Kinyarwanda (Rwanda and Burundi). The process for tool translation followed standard procedures and was described in detail^17^.

### Measurements

The outcome of interest in the current report is the perceived importance of future pandemic preparedness. The perceived importance of future pandemic preparedness was assessed using the above described 13-item questionnaire. Each question had five-point Likert scale answer options with scores 1 to 5 for not important, somewhat important, important, very important, and extremely important, respectively. The higher scores indicated higher perceived importance of that specific item for future pandemic preparedness. We treated each item individually rather than calculating a total or sub-totals of perceived future pandemic preparedness. The above 13 items can be loosely grouped into four dimensions: online medical consultations (#1–3), online learning (#4–5), messaging for health information (#6–9), and internet-facilitated shopping (#10–13).

The exposure of interest is e-Health literacy. e-Health literacy was measured using the e-Health literacy scale (e-HEALS), which was validated in English with a reliability coefficient (Cronbach’s alpha) of 0.88 ^18^. In the current sample, we recorded very high reliability coefficients for the e-HEALS (the overall Cronbach’s alpha of 0.93 and the same coefficient among Kinyarwanda respondents). The response options for each of the above described 8 e-HEALS items is a five-point Likert Scale ranging from “strongly disagree” (1) to “strongly agree” (5). Total e-Health literacy score is calculated by summing the individual scores of each question, and the maximum possible total score is 40. We treated e-Health literacy scores as a continuous variable.

### Covariates

The covariates in this study were the participants’ characteristics: age, gender, marital status, education, occupation, perceived social rank, and country of residence. Age was measured as a categorical variable and was coded as 18–29, 30–39, and ≥ 40 years. In addition, gender was a categorical variable classified as male, female, or non-binary. The marital status was treated as a categorical variable, and it was recorded as married/cohabitation/common-law, separated/divorced/widowed, and single. Education was also measured as an ordinal variable collected as primary, secondary, postsecondary, and graduate education (e.g., master’s, doctorate, Medical Doctor). The occupation variable was measured as a binary variable collected as either employed or unemployed. Perceived social rank was self-reported by participants using an ordinal variable ranging from 1 to 5.

### Statistical analysis

We summarised the unweighted and weighted participants’ age and gender characteristics by country of residence. We used mean and standard deviation or standard error for continuous variables and frequencies and percentages for the categorical variables. We used country weights based on countries’ population, age, and gender distribution. We performed the confirmatory factor analysis of the e-HEALS and the perceived importance of pandemic preparedness questionnaire to confirm the invariance of responses recorded with both English and Kinyarwanda questionnaires (Supplementary File 1). To explore the association between e-Health literacy and the perceived importance of future pandemic preparedness, we conducted the unadjusted logistic regression model between the total eHealth literacy score and each question of the perceived future pandemic preparedness questionnaire. We then repeated the model, adjusting for age, gender, marital status, education, occupation, perceived social rank, and country of residence. All significance tests were two-sided and used 5% nominal level of significance. We used Stata software version BE 17 for the data analysis.

### Ethical considerations

This online survey was carried out following online research guidelines and policy, and the ethical approval was obtained from the institutional review board of The University of Hong Kong—the Hospital Authority Hong Kong West Cluster (approval UW 20–272) and each participating country following local ethical regulation, with approval from College of Medicine and Health Sciences institutional review board in Rwanda (No 330/CMHS IRB/2020). An informed consent was obtained from all participants and the participation was voluntary, which was ensured by asking participants to confirm that they understood the terms of the informed consent by checking a box online. The research team additionally complied with all ethical requirements by anonymising collected data to ensure that confidentiality was safeguarded.

## Results

### Sociodemographic characteristics of respondents

A total of 1295 people from Burundi (*n* = 369), Nigeria (*n* = 587), Rwanda (*n* = 143), and South Africa (*n* = 196) participated in this study. Most participants, 685 (52.90%), were aged between 18 and 29 (Table 1). Roughly, 591 (45.64%) were males and over half of the participants were female, 685 (52.90%), single, 737 (56.91%), and had a higher education, 738 (57.04%). Most participants were employed, 786 (60.69%), and on a self-reported perceived social rank ranging from 1 (very poor) to 5 (very rich), roughly 80% reported to be ≤ average (3). The unstandardised average (SE) e-Health literacy score was 29.55 (0.19) out of 40. The weighted participants’ characteristics are provided (Supplementary File 1 – Table 1). In the standardized averages, Burundi had the lowest, and South Africa had the highest e-Health literacy scores.

**Table 1:** Sociodemographic characteristics of respondents (unweighted).

	Burundi	Nigeria	Rwanda	South Africa	Total
N	369	587	143	196	1295
**Age**					
18–29	85 (23.04%)	423 (72.06%)	59 (41.26%)	118 (60.20%)	685 (52.90%)
30–39	147 (39.84%)	119 (20.27%)	65 (45.45%)	37 (18.88%)	368 (28.41%)
>=40	137 (37.13%)	45 (7.67%)	19 (13.29%)	41 (20.92%)	242 (18.69%)
**Gender**					
Female	181 (49.05%)	329 (56.05%)	40 (27.97%)	135 (68.88%)	685 (52.90%)
Male	177 (47.97%)	254 (43.27%)	100 (69.93%)	60 (30.61%)	591 (45.64%)
Non-binary	11 (2.98%)	4 (0.68%)	3 (2.10%)	1 (0.51%)	19 (1.47%)
**Marital Status**					
Married/Cohabitation	236 (63.96%)	156 (26.58%)	66 (46.15%)	67 (34.18%)	525 (40.54%)
Separated/Divorced/Widow	14 (3.79%)	7 (1.19%)	5 (3.50%)	7 (3.57%)	33 (2.55%)
Single	119 (32.25%)	424 (72.23%)	72 (50.35%)	122 (62.24%)	737 (56.91%)
**Education**					
<=Primary	139 (37.67%)	3 (0.51%)	0 (0.00%)	0 (0.00%)	142 (10.97%)
Secondary	180 (48.78%)	28 (4.77%)	23 (16.20%)	35 (17.86%)	266 (20.56%)
Higher education	46 (12.46%)	484 (82.46%)	80 (56.34%)	128 (65.30%)	738 (57.04%)
>=Graduate	4 (1.08%)	72 (12.27%)	39 (27.46%)	33 (16.84%)	148 (11.44%)
**Employment status**					
Unemployed	92 (24.93%)	275 (46.85%)	34 (23.78%)	108 (55.10%)	509 (39.31%)
Employed	277 (75.07%)	312 (53.15%)	109 (76.22%)	88 (44.90%)	786 (60.69%)
**Perceived social rank**					
1	165 (44.72%)	18 (3.07%)	4 (2.80%)	15 (7.65%)	202 (15.60%)
2	169 (45.80%)	151 (25.72%)	38 (26.57%)	24 (12.24%)	382 (29.50%)
3	30 (8.13%)	273 (46.51%)	81 (56.64%)	83 (42.35%)	467 (36.06%)
4	5 (1.36%)	110 (18.74%)	18 (12.59%)	57 (29.08%)	190 (14.67%)
5	0 (0.00%)	35 (5.96%)	2 (1.40%)	17 (8.67%)	54 (4.17%)
**E-Health Scores (ranges from 5–40)**, **mean (SE) (***n*** = 1295)**	26.34 (0.36)	30.83 (0.26)	31.73 (0.50)	30.18 (0.41)	29.55 (0.19)

### Perceived importance of future pandemic preparedness

**Fig. 1:**
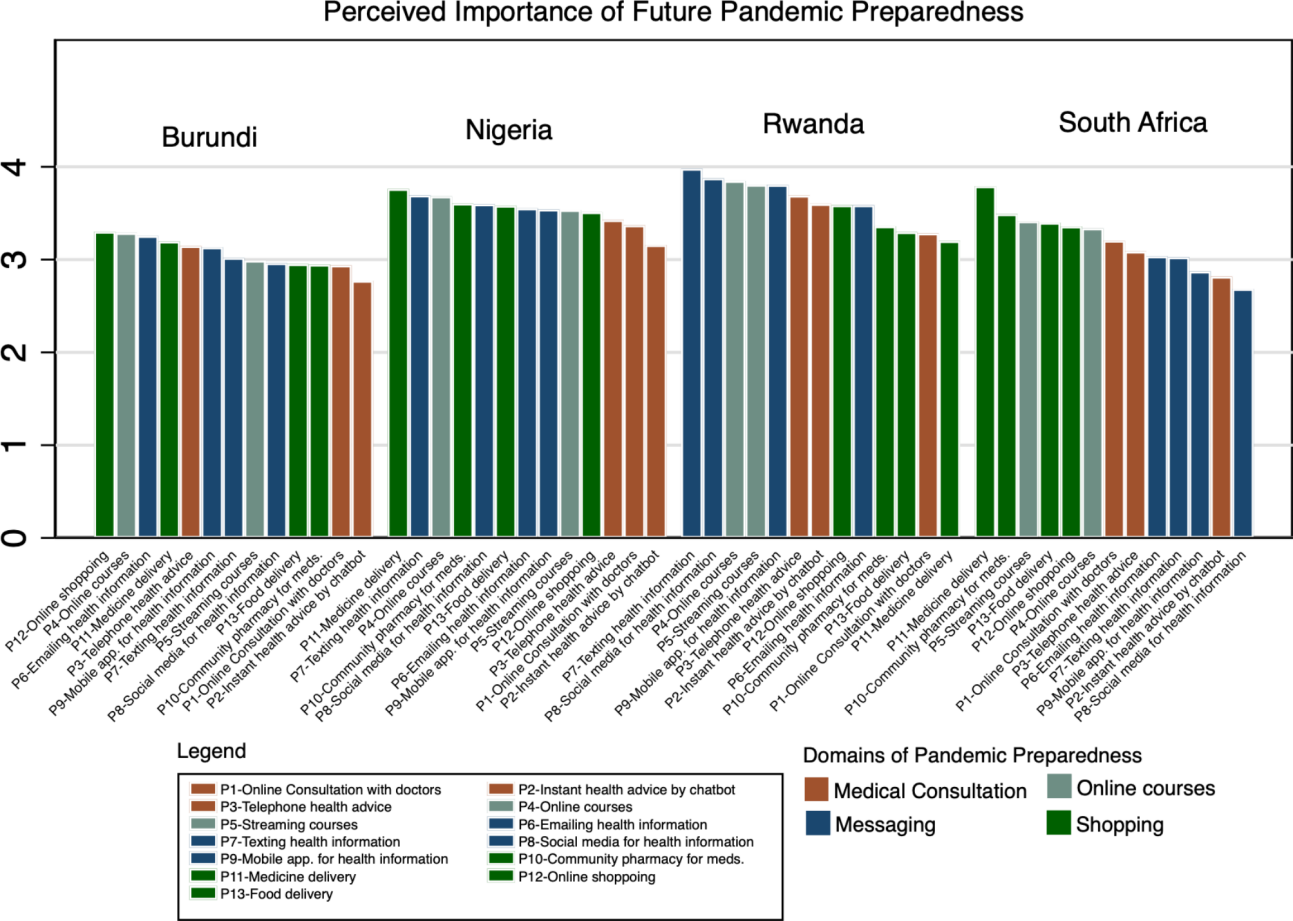
Perceived importance of future pandemic preparedness by the country.

Across countries, shopping facilitated with internet was the most perceived as an important dimension of future pandemic readiness, except in Rwanda (Fig. 1). On the contrary, online medical consultation as a key dimension of future pandemic preparedness was predominantly perceived as a less important element across participating countries, particularly in Nigeria and Burundi (Check supplementary File 1 – Table 2 for weighted averages of perceived importance of pandemic preparedness scores and associated standard errors). While messaging for health information was perceived as the most important dimension of pandemic preparedness in Rwanda (mean: 4.07 (SE: 0.12)), it was the least important dimension in South Africa (mean: 2.82 (SE:0.14)). Online courses were perceived as a moderately important readiness dimension of future pandemic preparedness across all countries.

### Association between e-Health literacy and perceived importance of future pandemic preparedness

e-Health literacy was positively associated with 8 of 13 preparedness variables in the adjusted logistic regression model (Table 2). Two elements of the medical consultation dimension of future pandemic preparedness were associated with e-Health literacy, online consultation with doctors (OR = 1.11, 95% CI 1.02–1.21) and telephone health advice (OR = 1.07, 95% CI 1.00–1.15), respectively. Every unit increase in e-Health literacy was associated with 11% higher odds of perceiving online consultation as a more important element of pandemic preparedness. Instant streaming courses, an element of online course dimension, was associated with e-Health literacy (OR = 1.09, 95% CI 1.02–1.16). Of the four elements in the dimension of messaging for health information, only two were associated with e-Health literacy, and these were: receiving health information via email (OR = 1.08, 95% CI 1.01–1.17) and via social media (e.g. Facebook, Instagram, Twitter (currently known as X) (OR = 1.08, 95% CI 1.03–1.14). Three of the four elements of the internet-facilitated shopping for medications and other supplies dimension were associated with e-Health literacy, and these were: getting medicine prescribed in a hospital visit/follow-up in a community pharmacy (OR = 1.07, 95% CI 1.05–1.10), medicine delivery (OR = 1.04, 95% CI 1.03–1.06), and online shopping (OR = 1.07, 95% CI 1.03–1.11). Country specific unadjusted and adjusted results of logistic regression models are also reported (Supplementary File 2- The country specific analysis).

**Table 2:** Unadjusted and adjusted results of logistic regression models between e-Health literacy and perceived importance of future pandemic preparedness (*n* = 1295).

	Unadjusted, OR 95% CI	Adjusted, OR; 95% CI^1^
**Medical consultation by use of the Internet or phone**		
a. Online consultation with doctors (e.g. Zoom, Skype)	**1.12 (1.05**, 1.19) *	**1.11 (1.02**, 1.21) *
b. Instant personalised health advice by online chatbot	1.08 (0.97, 1.20)	1.08 (0.99, 1.17)
c. Telephone health advice	1.08 (0.98, 1.18)	**1.07 (1.00**, 1.15) *
**Online courses**		
d. Online courses	1.09 (1.00, 1.19)	1.08 (0.99, 1.18)
e. Instant streaming courses (e.g. Zoom, Skype)	**1.10 (1.01**, 1.21) *	**1.09 (1.02**, 1.16) *
**Messaging for health information**		
f. Receiving health information through email	1.07 (0.98, 1.18)	**1.08 (1.01**, 1.17) *
g. Receiving health information through text messaging (e.g. SMS, WhatsApp)	1.10 (0.94, 1.28)	1.09 (0.98, 1.21)
h. Receiving health information from social media (e.g. Facebook, Instagram, Twitter)	1.09 (0.99, 1.20)	**1.08 (1.03**, 1.14) *
i. Receiving health information from a mobile app	1.10 (0.97, 1.25)	1.09 (0.99, 1.20)
**Internet facilitated shopping for medications and other supplies**		
j. Get medicine prescribed in a hospital visit/follow-up in a community pharmacy	**1.09 (1.02**, 1.16) *	**1.07 (1.05**, 1.10) **
k. Medicine delivery	**1.06 (1.04**, 1.07) **	**1.04 (1.03**, 1.06) **
l. Online shopping	**1.07 (1.02**,** 1.12)**	**1.07 (1.03**, 1.11) **
m. Food delivery	1.07 (0.94, 1.22)	1.07 (0.96, 1.18)

Statistically significant: *p-value < 0.05, **p-value < 0.01.

^1^Adjusted for age, gender, marital status, education, occupation, perceived social rank, and country of residence.

## Discussion

### Principal findings

To the best of our knowledge, this study is the first to investigate the association between e-Health literacy and the perception of future pandemic preparedness. The mean (SE) of e-HEALS was 29.55 (0.19). SSA countries had different perceptions of what elements are important for future pandemic preparedness, and we found that e-Health literacy was associated with most elements we explored being perceived as important for future pandemic preparedness.

### e-Health literacy

The standardised e-HEALS showed a total mean of 29.55 (SE = 0.19). The results revealed that Burundi had the least and South Africa had the highest mean scores of e-Health literacy. The disparity in e-Health literacy across countries may be attributed to the countries’ digital profile, with internet penetration being only 5.23% in Burundi as of 2021 when this study was being carried out, whereas it was 74.17% in South Africa the same year^19,20^. Additionally, Burundi was classified as the least connected country worldwide in 2021, using various criteria including but not limited to the rate of individuals with mobile, internet and broadband access, mean internet download and upload speeds and internet affordability^21^. The lack of digital devices, which was linked to Burundi’s low acceptability of health interventions, can also be linked to the lowest level of e-Health literacy or limits the use of preventative interventions, thus resulting in poor health outcomes^22^. Other factors such as unemployment status, low literacy, being a woman and old person were documented to hamper the use of internet in SSA^23^.

### The perception of future pandemic preparedness

Internet-facilitated shopping, which was deemed as the most significant aspect of future pandemic preparedness across all participating countries apart from Rwanda, had received a higher perception in South Africa. South African participants put forward preparing for future pandemics by facilitating online shopping and medicine delivery. We feel that this correlates with the recent COVID-19 pandemic preparedness measure devised by the South African government via the Metropolitan Health Services to order online, collect and deliver medication to vulnerable populations using the available transport system in the country alongside volunteers^24^. An additional factor to that measure is the daily load-shedding implemented in South Africa that has made in-store purchasing more inconvenient, thus driving numerous people to shop online^25^.The advantages of this newly implemented system have been significant, from decreasing the spread of COVID-19 among vulnerable populations and improving relationships between health facility staff and community healthcare workers to delivering other essential items purchased online^24^.

However, it is undeniable that this practice may be difficult in most SSA countries due to the low rate of internet use compounded by transport issues^21,23^. The scarce access to the internet has an implication in ordering the needed items, thus making it hard to shop online for medicine and other essential needs, needless to mention their delivery. Although currently evolving, online banking or the use of bank ATM cards is also not yet sufficiently common in some SSA countries, thus hampering online shopping or ordering. Notably, the online banking penetration rate in Africa is estimated to be 8.01% in 2024 ^26^. For example, except South Africa that has bank account penetration and online banking penetration of 87.89% and 51.69% in 2024 ^27^, while other countries like Rwanda, Nigeria and Burundi have a relatively low bank account penetration (and a very low online banking penetration), with 66.07% (3.23%), 58.30% (4.30%), and 18.53% (1.91%), respectively^28–30^. This shows that online shopping of both items is deemed hard in SSA countries, underscoring the need to improve the continent’s banking system.

Receiving health information through text messaging and mobile apps was the most preferred preparedness in Rwanda, where the RapidSMS system is commonly used. This data correlates with the findings of a comparative study between healthcare facilities using RapidSMS and others that did not, revealing the increasing postnatal care visit rates among women and a drop in the trend of malnutrition in districts using the RapidSMS system^31^. The use of messaging for health intervention was also significant in Rwanda’s Home-Based care in the context of COVID-19 preventive policies in informing timely public health guidance and policies^32^. This underscores how receiving adequate health information via messaging is crucial in treating and preventing it as it can improve understanding of necessary behaviour to tackle infectious diseases^33^.

It’s interesting to note that while online medical consultation is an essential component of future pandemic preparedness, it was viewed as less significant in participating nations, especially in Nigeria and Burundi. This may be linked to the lack of online medical services in many SSAs on one hand. On the other hand, the low e-Health literacy, along with the limited internet services^21,23^ may be factors that lead to low perception of online medical consultation in SSA. Despite the low perception, we argue that fostering online medical consultation can increase access to convenient healthcare services.

### Association between e-Health literacy and perceived importance of future pandemic preparedness

People’s behaviours towards the readiness for any potential disaster are dictated by their risk perception, meaning that what people perceive can reflect a better emergency preparedness. In the current study, we found that the higher e-Health literacy was associated with dimensions and most items we explored were perceived as important in pandemic preparedness. It is, however, important to note that the direction of association can go both ways. For instance, people who perceive online messaging and doctor consultation to be important elements of pandemic preparedness could wind up investing more in their e-Health literacy. Previous studies have also linked high e-Health literacy with compliance to infection and prevention control measures^34^, something which can come handy in emerging and re-emerging infectious diseases pandemic response. A previous study reported that exposure to health information through news, social media, and health websites did not necessarily increase COVID-19 risk perception^35^. Multiple studies noted that e-Health literacy, coupled with receiving appropriate health information, improved the population’s uptake of COVID-19 testing and adherence to devised protective measures^11,13,36^. Whatever the direction of the association, the improvement of e-Health literacy could be useful in emerging and re-emerging infectious diseases response efforts through people becoming more aware of the ways by which computer and internet usage can be useful in preparing and responding to an EID. One of the ways to improve e-Health should be ensuring the affordability and availability of electronic devices and internet among population in financial constraints. Additionally, health intervention strategies customized in targeted population’s first language can improve e-Health literacy in relevance to language proficiency essential for health literacy^37^.

### Limitations

This study had some limitations. The questionnaire was self-administered, thus increasing the risk of social desirability bias. Similarly, some questions may not have been fully understood, but this was anticipated by pilot testing the questionnaire to ensure it was accepted by the local population before it was fully rolled out, and by testing the invariance of languages used. Another equally important factor that could have constituted a limitation could be the limited access to phones and/ internet given that an important number of rural dwellers who constitute the overwhelming majority of the population from the above-mentioned countries could not have had accessed online questionnaire. There is also a probability that the majority of the participants could have been like-minded people who recruited one another, thus influencing the results. Equally, we recognise that the same respondents may have completed and submitted the same questionnaires more than once, however, this was addressed by eliminating redundant responses.

Further, we conducted a cross-sectional observational study; hence, we could not produce evidence about causality in the relationship between e-Health literacy and perceived importance of future pandemic preparedness. A future longitudinal study may best serve this role. Finally, the use of convenient sampling methods suggests the limitations in generalising the results as the method can influence the underrepresentation of less privileged individuals without accessibility to digital tools, with low digital literacy and lack of internet. Fortunately, weighted analyses were performed to ensure completeness of the data.

## Conclusion

This study reported the association between e-Health literacy and the perception of future outbreaks and pandemic preparations. The e-Health literacy and perception regarding preparedness for future pandemic preparedness varied among the participating countries in SSA. Higher level of e-Health literacy was associated with higher perception of all dimensions we explored as important for future pandemics’ readiness. Bolstering e-Health literacy may increase the efficiency of future pandemic preparedness in SSA. Further studies should explore the driving factors of low perceived importance of online medical consultation in SSA.

## Electronic supplementary material

Below is the link to the electronic supplementary material.

Supplementary Material 1

Supplementary Material 2

## Data Availability

Data are available to the corresponding author on request.
