# Supplementary material for: Association between e-health literacy and perceived importance of future pandemic preparedness in sub-saharan Africa
Source: Sci Rep. 2024 Dec 28;14:30734. doi: 10.1038/s41598-024-80121-x (PMC11681008; doi:10.1038/s41598-024-80121-x)
Supplement: Supplementary file 1 — Supplementary Material 1 [file 41598_2024_80121_MOESM1_ESM.docx]

**Table 1. Weighted Participants Characteristics reported in n (**%) (n=1295)

|  | **Burundi** | **Nigeria** | **Rwanda** | **South Africa** | **Total** |
| --- | --- | --- | --- | --- | --- |
| N | 369 | 587 | 143 | 196 | 1295 |
| **Age** |  |  |  |  |  |
| 18-29 | 150 (40.61%) | 245 (41.72%) | 57 (40.12%) | 60 (30.66%) | 512 (39.57%) |
| 30-39 | 93 (25.15%) | 162 (27.56%) | 47 (33.09%) | 62 (31.63%) | 363 (28.05%) |
| >=40 | 126 (34.24%) | 180 (30.72%) | 38 (26.79%) | 74 (37.71%) | 419 (32.38%) |
|  |  |  |  |  |  |
| **Gender** |  |  |  |  |  |
| Female | 178 (48.37%) | 290 (49.46%) | 69 (48.22%) | 97 (49.52%) | 635 (49.02%) |
| Male | 172 (46.66%) | 294 (50.09%) | 69 (48.16%) | 99 (50.38%) | 634 (48.94%) |
| Non-binary | 18 (4.96%) | 3 (0.45%) | 5 (3.62%) | 0 (0.10%) | 26 (2.04%) |
|  |  |  |  |  |  |
| **Marital Status** |  |  |  |  |  |
| Married | 190 (51.41%) | 283 (48.19%) | 73 (51.12%) | 109 (55.64%) | 655 (50.55%) |
| Separated | 24 (6.45%) | 21 (3.55%) | 8 (5.79%) | 9 (4.70%) | 62 (4.80%) |
| Single | 155 (42.13%) | 283 (48.27%) | 62 (43.09%) | 78 (39.66%) | 578 (44.65%) |
|  |  |  |  |  |  |
| **Education** |  |  |  |  |  |
| <=Primary | 133 (36.17%) | 2 (0.36%) | 0 (0.00%) | 0 (0.00%) | 138 (10.66%) |
| Secondary | 178 (48.18%) | 23 (3.92%) | 32 (22.72%) | 27 (13.90%) | 262 (20.24%) |
| Higher education | 52 (13.97%) | 413 (70.28%) | 67 (46.94%) | 116 (59.04%) | 645 (49.79%) |
| >=Graduate | 6 (1.68%) | 149 (25.44%) | 43 (30.35%) | 53 (27.06%) | 250 (19.30%) |
|  |  |  |  |  |  |
| **Employment status** |  |  |  |  |  |
| Unemployed | 120 (32.59%) | 203 (34.61%) | 40 (27.67%) | 47 (24.13%) | 411 (31.72%) |
| Employed | 249 (67.41%) | 384 (65.39%) | 103 (72.33%) | 149 (75.87%) | 884 (68.28%) |
|  |  |  |  |  |  |
| **PSR** |  |  |  |  |  |
| 1 | 173 (46.76%) | 13 (2.20%) | 9 (6.04%) | 6 (3.29%) | 203 (15.70%) |
| 2 | 149 (40.28%) | 124 (21.18%) | 40 (27.76%) | 12 (5.94%) | 325 (25.12%) |
| 3 | 38 (10.25%) | 251 (42.70%) | 74 (52.02%) | 82 (41.78%) | 442 (34.12%) |
| 4 | 10 (2.70%) | 134 (22.86%) | 19 (13.39%) | 78 (39.66%) | 240 (18.55%) |
| 5 | 0 (0.00%) | 65 (11.05%) | 1 (0.80%) | 18 (9.33%) | 84 (6.51%) |
|  |  |  |  |  |  |
| **E-health (SE)** | 26.9 (0.5) | 30.6 (0.3) | 30.9 (0.6) | 31.1 (0.6) | 29.64 (0.25) |

**Table 2. Weighted Averages of Perceived Importance of Pandemic Preparedness Scores (Standard Error)**

|  | **Burundi,**  **Mean (SE)** | **Nigeria,**  **Mean (SE)** | **Rwanda,**  **Mean (SE)** | **South Africa,**  **Mean (SE)** |
| --- | --- | --- | --- | --- |
| **Medical consultation by use of the Internet or phone** |  |  |  |  |
| a. Online consultation with doctors (e.g. Zoom, Skype) | 3.03 (0.07) | 3.31 (0.07) | 3.31 (0.14) | 3.33 (0.12) |
| b. Instant personalised health advice by online chatbot | 2.88 (0.08) | 3.15 (0.07) | 3.81 (0.13) | 2.73 (0.15) |
| c. Telephone health advice | 3.18 (0.07) | 3.41 (0.07) | 3.83 (0.13) | 3.08 (0.13) |
| **Online courses** |  |  |  |  |
| d. Online courses | 3.32 (0.07) | 3.69 (0.06) | 3.88 (0.13) | 3.40 (0.13) |
| e. Instant streaming courses (e.g. Zoom, Skype) | 3.02 (0.08) | 3.54 (0.07) | 3.78 (0.15) | 3.40 (0.14) |
| **Messaging for health information** |  |  |  |  |
| f. Receiving health information through email | 3.33 (0.07) | 3.58 (0.06) | 3.47 (0.14) | 2.94 (0.14) |
| g. Receiving health information through text messaging (e.g. SMS, WhatsApp) | 3.10 (0.08) | 3.72 (0.06) | 4.07 (0.12) | 2.82 (0.14) |
| h. Receiving health information from social media (e.g. Facebook, Instagram, Twitter) | 3.01 (0.09) | 3.58 (0.06) | 3.91 (0.16) | 2.53 (0.16) |
| i. Receiving health information from a mobile app | 3.18 (0.07) | 3.52 (0.06) | 3.91 (0.14) | 2.69 (0.15) |
| **Internet facilitated shopping for medications and other supplies** |  |  |  |  |
| j. Get medicine prescribed in a hospital visit/follow-up in a community pharmacy | 3.01 (0.07) | 3.59 (0.06) | 3.22 (0.17) | 3.40 (0.13) |
| k. Medicine delivery | 3.23 (0.07) | 3.71 (0.06) | 3.15 (0.13) | 3.73 (0.12) |
| l. Online shopping | 3.32 (0.06) | 3.48 (0.06) | 3.55 (0.13) | 3.48 (0.14) |
| m. Food delivery | 3.01 (0.06) | 3.54 (0.06) | 3.21 (0.13) | 3.31 (0.16) |

**Table 3. Unweighted Prevalence of Dichotomized Perceived Importance of Pandemic Preparedness**

|  |  | **Burundi,**  **n (%)** | **Nigeria,**  **n (%)** | **Rwanda,**  **n (%)** | **South Africa,**  **n (%)** | **Total**  **n (%)** |
| --- | --- | --- | --- | --- | --- | --- |
| N |  | 369 | 587 | 143 | 196 | 1295 |
| **Medical consultation by use of the Internet or phone** |  |  |  |  |  |  |
| a. Online consultation with doctors (e.g. Zoom, Skype) | Less important | 138 (37.40%) | 125 (21.29%) | 21 (14.69%) | 41 (20.92%) | 325 (25.10%) |
|  | More important | 231 (62.60%) | 462 (78.71%) | 122 (85.31%) | 155 (79.08%) | 970 (74.90%) |
| b. Instant personalised health advice by online chatbot | Less important | 158 (42.82%) | 151 (25.72%) | 19 (13.29%) | 73 (37.24%) | 401 (30.97%) |
|  | More important | 211 (57.18%) | 436 (74.28%) | 124 (86.71%) | 123 (62.76%) | 894 (69.03%) |
| c. Telephone health advice | Less important | 98 (26.56%) | 99 (16.87%) | 13 (9.09%) | 56 (28.57%) | 266 (20.54%) |
|  | More important | 271 (73.44%) | 488 (83.13%) | 130 (90.91%) | 140 (71.43%) | 1029 (79.46%) |
| **Online courses** |  |  |  |  |  |  |
| d. Online courses | Less important | 76 (20.60%) | 68 (11.58%) | 10 (6.99%) | 45 (22.96%) | 199 (15.37%) |
|  | More important | 293 (79.40%) | 519 (88.42%) | 133 (93.01%) | 151 (77.04%) | 1096 (84.63%) |
| e. Instant streaming courses (e.g. Zoom, Skype) | Less important | 134 (36.31%) | 83 (14.14%) | 11 (7.69%) | 44 (22.45%) | 272 (21.00%) |
|  | More important | 235 (63.69%) | 504 (85.86%) | 132 (92.31%) | 152 (77.55%) | 1023 (79.00%) |
| **Messaging for health information** |  |  |  |  |  |  |
| f. Receiving health information through email | Less important | 85 (23.04%) | 73 (12.44%) | 16 (11.19%) | 56 (28.57%) | 230 (17.76%) |
|  | More important | 284 (76.96%) | 514 (87.56%) | 127 (88.81%) | 140 (71.43%) | 1065 (82.24%) |
| g. Receiving health information through text messaging (e.g. SMS, WhatsApp) | Less important | 136 (36.86%) | 64 (10.90%) | 13 (9.09%) | 62 (31.63%) | 275 (21.24%) |
|  | More important | 233 (63.14%) | 523 (89.10%) | 130 (90.91%) | 134 (68.37%) | 1020 (78.76%) |
| h. Receiving health information from social media (e.g. Facebook, Instagram, Twitter) | Less important | 143 (38.75%) | 82 (13.97%) | 20 (13.99%) | 83 (42.35%) | 328 (25.33%) |
|  | More important | 226 (61.25%) | 505 (86.03%) | 123 (86.01%) | 113 (57.65%) | 967 (74.67%) |
| i. Receiving health information from a mobile app | Less important | 112 (30.35%) | 80 (13.63%) | 15 (10.56%) | 72 (36.92%) | 279 (21.58%) |
|  | More important | 257 (69.65%) | 507 (86.37%) | 127 (89.44%) | 123 (63.08%) | 1014 (78.42%) |
| **Internet facilitated shopping for medications and other supplies** |  |  |  |  |  |  |
| j. Get medicine prescribed in a hospital visit/follow-up in a community pharmacy | Less important | 145 (39.30%) | 67 (11.41%) | 23 (16.08%) | 31 (15.82%) | 266 (20.54%) |
|  | More important | 224 (60.70%) | 520 (88.59%) | 120 (83.92%) | 165 (84.18%) | 1029 (79.46%) |
| k. Medicine delivery | Less important | 93 (25.20%) | 55 (9.37%) | 20 (13.99%) | 20 (10.20%) | 188 (14.52%) |
|  | More important | 276 (74.80%) | 532 (90.63%) | 123 (86.01%) | 176 (89.80%) | 1107 (85.48%) |
| l. Online shopping | Less important | 70 (18.97%) | 103 (17.55%) | 15 (10.49%) | 43 (21.94%) | 231 (17.84%) |
|  | More important | 299 (81.03%) | 484 (82.45%) | 128 (89.51%) | 153 (78.06%) | 1064 (82.16%) |
| m. Food delivery | Less important | 121 (32.79%) | 91 (15.50%) | 24 (16.78%) | 41 (20.92%) | 277 (21.39%) |
|  | More important | 248 (67.21%) | 496 (84.50%) | 119 (83.22%) | 155 (79.08%) | 1018 (78.61%) |

**E-health Psychometric Properties in English**

**Table 4. Goodness of fit test after structural equation modeling assuming one latent factor**

| **Fit statistic** | **Value** | **Description** |
| --- | --- | --- |
| **Likelihood ratio** |  |  |
| chi2_ms (20) | 328.532 | model vs. saturated |
| p > chi2 | <0.001 |  |
| chi2_bs (28) | 7050.286 | baseline vs. saturated |
| p > chi2 | <0.001 |  |
| **Population error** |  |  |
| RMSEA | 0.109 | Root mean squared error of approximation |
| 90% CI, lower bound | 0.099 |  |
| upper bound | 0.12 |  |
| pclose | <0.001 | Probability RMSEA <= 0.05 |
| **Information criteria** |  |  |
| AIC | 23185.026 | Akaike's information criterion |
| BIC | 23309.016 | Bayesian information criterion |
| **Baseline comparison** |  |  |
| CFI | 0.956 | Comparative fit index |
| TLI | 0.938 | Tucker Lewis index |
| **Size of residuals** |  |  |
| SRMR | 0.036 | Standardized root mean squared residual |
| CD | 0.933 | Coefficient of determination |

**Table 5. Reliability of e-Health survey on the whole sample**

| Item | Obs | Sign | Item-test correlation | Item-rest correlation | Average interitem covariance | alpha |
| --- | --- | --- | --- | --- | --- | --- |
| eheals1 | 1295 | + | 0.7758 | 0.7058 | 0.6642971 | 0.9215 |
| eheals2 | 1295 | + | 0.8254 | 0.7682 | 0.6478543 | 0.9169 |
| eheals3 | 1295 | + | 0.8461 | 0.7936 | 0.6381984 | 0.9149 |
| eheals4 | 1295 | + | 0.8507 | 0.7996 | 0.6367246 | 0.9145 |
| eheals5 | 1295 | + | 0.8483 | 0.7962 | 0.6365474 | 0.9147 |
| eheals6 | 1295 | + | 0.8161 | 0.7521 | 0.6414938 | 0.9181 |
| eheals7 | 1295 | + | 0.8031 | 0.7339 | 0.6431743 | 0.9195 |
| eheals8 | 1295 | + | 0.7594 | 0.6758 | 0.6545779 | 0.9243 |
| Test scale |  |  |  |  | 0.6453585 | 0.9276 |

**Figure 1. Scree plot after principal component analysis suggesting of one factor for all respondents.**


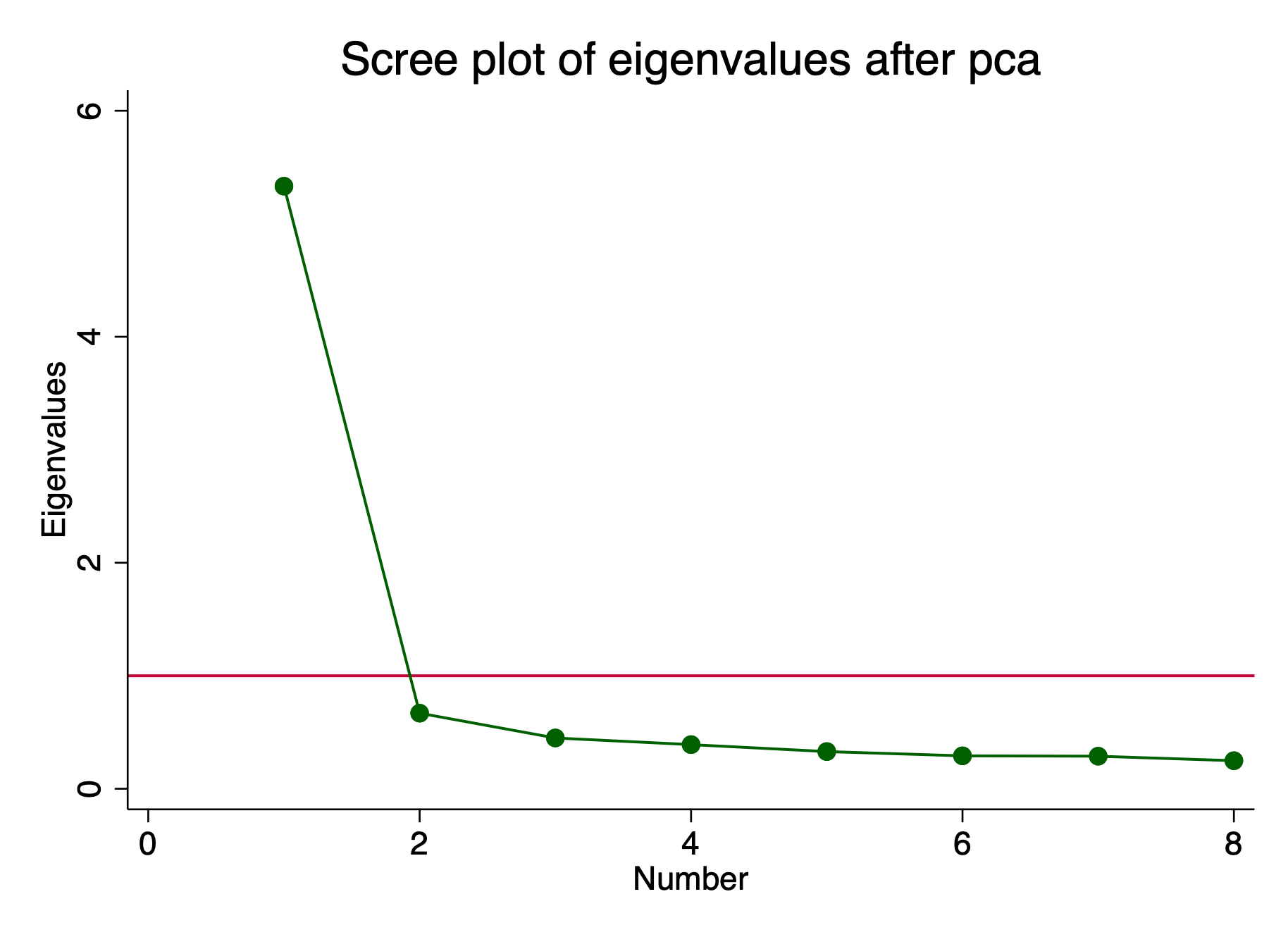


**E-health Psychometric Properties in Kinyarwanda**

**Table 6. Goodness of fit test after structural equation modeling assuming one latent factor for Kinyarwanda Respondents**

| **Fit statistic** | **Value** | **Description** |
| --- | --- | --- |
| **Likelihood ratio** |  |  |
| chi2_ms (20) | 113.888 | model vs. saturated |
| p > chi2 | <0.001 |  |
| chi2_bs (28) | 2627.818 | baseline vs. saturated |
| p > chi2 | <0.001 |  |
| **Population error** |  |  |
| RMSEA | 0.1 | Root mean squared error of approximation |
| 90% CI, lower bound | 0.083 |  |
| upper bound | 0.118 |  |
| pclose | <0.001 | Probability RMSEA <= 0.05 |
| **Information criteria** |  |  |
| AIC | 8732.962 | Akaike's information criterion |
| BIC | 8832.628 | Bayesian information criterion |
| **Baseline comparison** |  |  |
| CFI | 0.964 | Comparative fit index |
| TLI | 0.949 | Tucker Lewis index |
| **Size of residuals** |  |  |
| SRMR | 0.032 | Standardized root mean squared residual |
| CD | 0.934 | Coefficient of determination |

**Table 7. Reliability of e-health survey for Kinyarwanda Respondents (n=470)**

| Item | Obs | Sign | Item-test correlation | Item-rest correlation | Average interitem covariance | alpha |
| --- | --- | --- | --- | --- | --- | --- |
| eheals1 | 470 | + | 0.7713 | 0.704 | 0.7599123 | 0.9266 |
| eheals2 | 470 | + | 0.8513 | 0.8034 | 0.7292763 | 0.9196 |
| eheals3 | 470 | + | 0.8472 | 0.7949 | 0.7205402 | 0.9199 |
| eheals4 | 470 | + | 0.8381 | 0.7816 | 0.7195119 | 0.9209 |
| eheals5 | 470 | + | 0.8319 | 0.7723 | 0.7183166 | 0.9216 |
| eheals6 | 470 | + | 0.8161 | 0.7517 | 0.7241871 | 0.9232 |
| eheals7 | 470 | + | 0.8089 | 0.7426 | 0.7271003 | 0.9239 |
| eheals8 | 470 | + | 0.8147 | 0.7511 | 0.7273574 | 0.9232 |
| Test scale |  |  |  |  | 0.7282753 | 0.9314 |

**Figure 2. Scree plot after principal component analysis suggesting one factor for Kinyarwanda respondents**


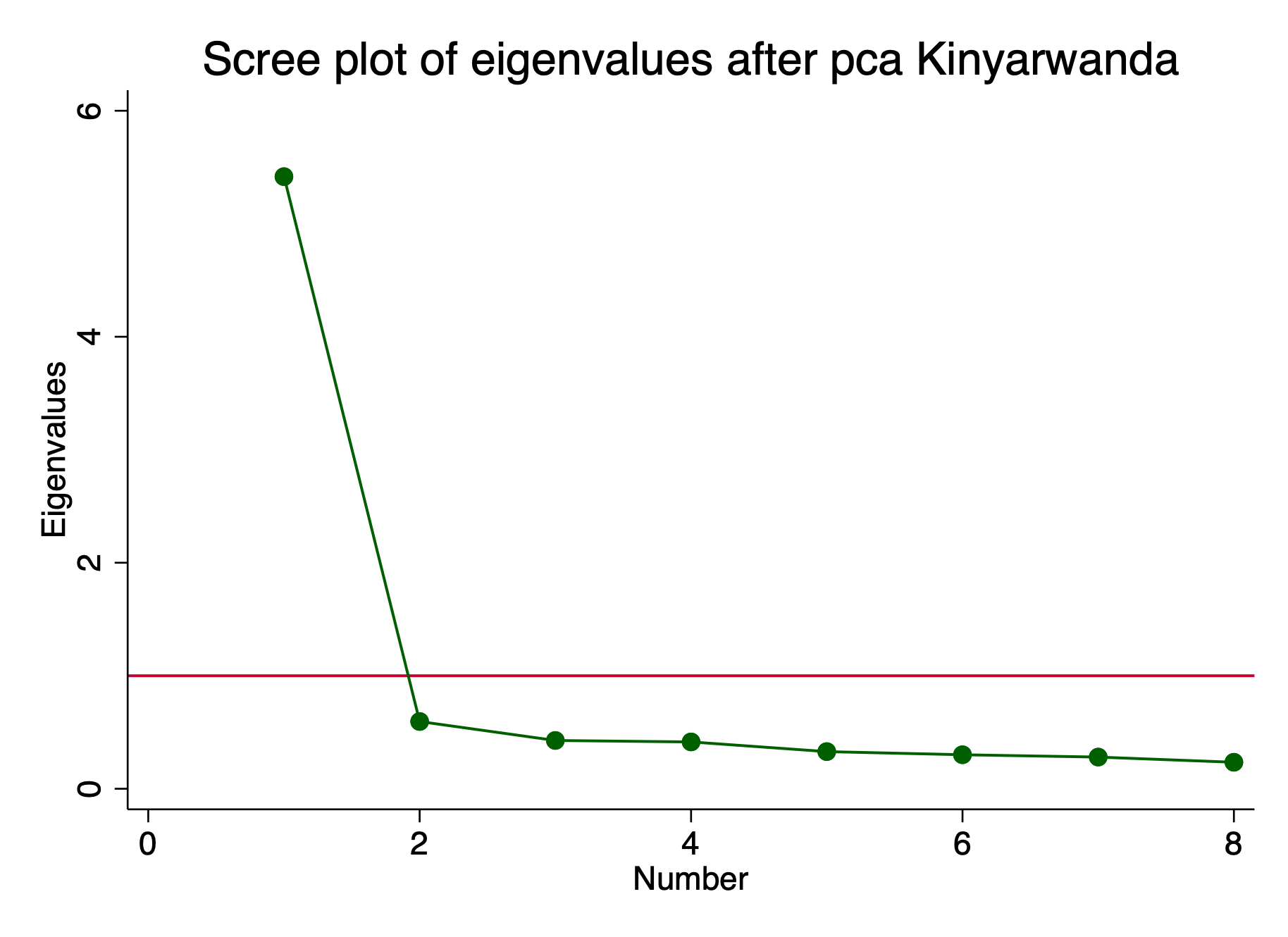


**Perceived Importance of Pandemic Preparedness Questionnaire Psychometric Properties for whole sample**

**Table 8. Reliability of perceived importance of pandemic preparedness questionnaire for all respondents**

| Item | Obs | Sign | Item-test correlation | Item-rest correlation | Average interitem covariance | alpha |
| --- | --- | --- | --- | --- | --- | --- |
| prepare1 | 1297 | + | 0.6736 | 0.6083 | 0.557463 | 0.9122 |
| prepare2 | 1297 | + | 0.7108 | 0.6471 | 0.5464586 | 0.9107 |
| prepare3 | 1297 | + | 0.7443 | 0.6916 | 0.5478741 | 0.909 |
| prepare4 | 1297 | + | 0.7318 | 0.678 | 0.5511946 | 0.9096 |
| prepare5 | 1297 | + | 0.7562 | 0.7028 | 0.5417312 | 0.9085 |
| prepare6 | 1297 | + | 0.7555 | 0.7062 | 0.5483595 | 0.9086 |
| prepare7 | 1297 | + | 0.7511 | 0.6955 | 0.5409015 | 0.9087 |
| prepare8 | 1297 | + | 0.7348 | 0.6732 | 0.5395431 | 0.9097 |
| prepare9_ | 1295 | + | 0.7328 | 0.6762 | 0.54729 | 0.9096 |
| prepare10 | 1297 | + | 0.6814 | 0.6173 | 0.5562693 | 0.9119 |
| prepare11 | 1297 | + | 0.6535 | 0.5876 | 0.5631481 | 0.9129 |
| prepare12 | 1297 | + | 0.6496 | 0.5799 | 0.5606051 | 0.9133 |
| prepare13 | 1297 | + | 0.6371 | 0.5615 | 0.5591883 | 0.9142 |
| Test scale |  |  |  |  | 0.5507712 | 0.917 |

**Perceived Importance of Pandemic Preparedness Questionnaire Psychometric Properties in Kinyarwanda**

**Table 9. Reliability of perceived importance of pandemic preparedness questionnaire for Kinyarwanda Respondents (n=471)**

| Item | Obs | Sign | Item-test correlation | Item-rest correlation | Average interitem covariance | alpha |
| --- | --- | --- | --- | --- | --- | --- |
| prepare1 | 471 | + | 0.6244 | 0.5546 | 0.519925 | 0.912 |
| prepare2 | 471 | + | 0.7122 | 0.6466 | 0.4978005 | 0.9085 |
| prepare3 | 471 | + | 0.7517 | 0.6996 | 0.4995216 | 0.9064 |
| prepare4 | 471 | + | 0.7488 | 0.6983 | 0.5029189 | 0.9066 |
| prepare5 | 471 | + | 0.7926 | 0.7436 | 0.486594 | 0.9044 |
| prepare6 | 471 | + | 0.7543 | 0.7046 | 0.5020236 | 0.9063 |
| prepare7 | 471 | + | 0.7276 | 0.6614 | 0.4915042 | 0.908 |
| prepare8 | 471 | + | 0.7697 | 0.7111 | 0.4832648 | 0.9057 |
| prepare9_ | 471 | + | 0.7423 | 0.6853 | 0.4966976 | 0.9069 |
| prepare10 | 471 | + | 0.6751 | 0.6092 | 0.5095346 | 0.9099 |
| prepare11 | 471 | + | 0.5395 | 0.4635 | 0.5346161 | 0.9151 |
| prepare12 | 471 | + | 0.6776 | 0.6185 | 0.5155267 | 0.9096 |
| prepare13 | 471 | + | 0.6238 | 0.5518 | 0.5183572 | 0.9121 |
| Test scale |  |  |  |  | 0.5044834 | 0.9151 |
