# Supplementary material for: Association between e-health literacy and perceived importance of future pandemic preparedness in sub-saharan Africa
Source: Sci Rep. 2024 Dec 28;14:30734. doi: 10.1038/s41598-024-80121-x (PMC11681008; doi:10.1038/s41598-024-80121-x)
Supplement: Supplementary file 2 — Supplementary Material 2 [file 41598_2024_80121_MOESM2_ESM.docx]

**Unadjusted and adjusted results of logistic regression models between e-Health literacy and perceived importance of future pandemic preparedness (n=1295)**

|  | **Unadjusted, OR 95% CI** | | | | **Fully Adjusted, OR 95% CI^1^** | | | |
| --- | --- | --- | --- | --- | --- | --- | --- | --- |
|  | **Rwanda (n=143)** | **Burundi (n=369)** | **Nigeria (n=587)** | **South Africa (n=196)** | **Rwanda (n=143)** | **Burundi (n=369)** | **Nigeria (n=587)** | **South Africa (n=196)** |
| prepare 1 | **1.09 (1.01, 1.18)** | **1.12 (1.08, 1.16)** | **1.13 (1.09, 1.16)** | 0.98 (0.93, 1.05) | **1.11 (1.02, 1.21)** | **1.10 (1.06, 1.14)** | **1.13 (1.09, 1.17)** | 0.99 (0.92, 1.05) |
| prepare 2 | 0.97 (0.89, 1.06) | **1.11 (1.07, 1.15)** | **1.09 (1.06, 1.13)** | 0.97 (0.92, 1.02) | 1.00 (0.92, 1.09) | **1.08 (1.04, 1.12)** | **1.10 (1.07, 1.14)** | 0.99 (0.93, 1.05) |
| prepare 3 | 0.97 (0.88, 1.08) | **1.12 (1.08, 1.16)** | **1.07 (1.03, 1.10)** | **1.01 (0.96, 1.07)** | 0.98 (0.88, 1.09) | **1.11 (1.06, 1.15)** | **1.07 (1.03, 1.11)** | 1.01 (0.96, 1.07) |
| prepare 4 | **1.14 (1.03, 1.27)** | **1.08 (1.04, 1.12)** | **1.11 (1.07, 1.15)** | **1.00 (0.95, 1.06)** | **1.22 (1.06, 1.40)** | **1.06 (1.01, 1.10)** | **1.10 (1.06, 1.15)** | 1.01 (0.95, 1.07) |
| prepare 5 | **1.12 (1.01, 1.24)** | **1.11 (1.07, 1.15)** | **1.10 (1.06, 1.14)** | 0.98 (0.93, 1.04) | 1.10 (0.98, 1.24) | **1.09 (1.05, 1.14)** | **1.10 (1.06, 1.14)** | 0.99 (0.93, 1.06) |
| prepare 6 | **1.10 (1.01, 1.20)** | **1.12 (1.07, 1.17)** | **1.06 (1.02, 1.10)** | **1.00 (0.95, 1.06)** | **1.10 (1.01, 1.21)** | **1.10 (1.05, 1.15)** | **1.06 (1.02, 1.10)** | 1.01 (0.95, 1.07) |
| prepare 7 | 1.03 (0.94, 1.13) | **1.14 (1.10, 1.19)** | **1.06 (1.02, 1.10)** | **1.01 (0.96, 1.07)** | 1.05 (0.95, 1.16) | **1.13 (1.08, 1.17)** | **1.06 (1.02, 1.10)** | 1.04 (0.98, 1.10) |
| prepare 8 | 1.01 (0.93, 1.09) | **1.14 (1.10, 1.19)** | **1.07 (1.03, 1.10)** | **1.01 (0.96, 1.06)** | 1.03 (0.94, 1.12) | **1.12 (1.08, 1.17)** | **1.07 (1.03, 1.11)** | 1.01 (0.95, 1.06) |
| prepare 9 | 0.99 (0.90, 1.08) | **1.14 (1.09, 1.18)** | **1.05 (1.01, 1.09)** | 0.98 (0.93, 1.03) | 1.00 (0.91, 1.10) | **1.11 (1.06, 1.15)** | **1.05 (1.01, 1.09)** | 0.99 (0.93, 1.04) |
| prepare 10 | 1.03 (0.96, 1.11) | **1.14 (1.10, 1.19)** | **1.06 (1.02, 1.10)** | **1.04 (0.98, 1.11)** | 1.02 (0.94, 1.11) | **1.12 (1.08, 1.17)** | **1.05 (1.01, 1.10)** | **1.07 (1.00, 1.15)** |
| prepare 11 | 1.04 (0.97, 1.13) | **1.07 (1.04, 1.11)** | 1.01 (0.96, 1.05) | **1.06 (0.99, 1.14)** | 1.05 (0.96, 1.14) | **1.06 (1.02, 1.10)** | 1.01 (0.96, 1.06) | 1.08 (0.99, 1.19) |
| prepare 12 | **1.10 (1.01, 1.20)** | **1.11 (1.07, 1.16)** | **1.07 (1.04, 1.11)** | **1.03 (0.98, 1.10)** | 1.09 (0.99, 1.20) | **1.09 (1.04, 1.14)** | **1.07 (1.03, 1.10)** | 1.03 (0.97, 1.10) |
| prepare 13 | 1.03 (0.96, 1.10) | **1.17 (1.12, 1.22)** | 1.02 (0.99, 1.06) | **1.03 (0.97, 1.09)** | 1.01 (0.93, 1.10) | **1.15 (1.10, 1.21)** | 1.02 (0.98, 1.06) | 1.03 (0.97, 1.10) |

Statistically significant: **Bolded**

^1^Adjusted for age, gender, marital status, education, occupation, perceived social rank.

prepare 1: Online consultation with doctors (e.g. Zoom, Skype)

prepare 2: Instant personalized health advice by online chatbot

prepare 3: Telephone health advice

prepare 4: Online courses

prepare 5: Instant streaming courses (e.g. Zoom, Skype)

prepare 6: Receiving health information through email

prepare 7: Receiving health information through text messaging (e.g. SMS, WhatsApp)

prepare 8: Receiving health information from social media (e.g. Facebook, Instagram, Twitter)

prepare 9: Receiving health information from a mobile app

prepare 10: Get medicine prescribed in a hospital visit/follow-up in a community pharmacy

prepare 11: Medicine delivery

prepare 12: Online shopping

prepare 13: Food delivery
